# Revising the Diabetes Distress Scale for Use Among Adults in the Dominican Republic: Findings From Cognitive Interviews

**DOI:** 10.1177/26350106221128003

**Published:** 2022-10-11

**Authors:** Deshira D. Wallace, Ivania Núñez, Clare Barrington

**Affiliations:** Department of Health Behavior, UNC Gillings School of Global Public Health, Chapel Hill, North Carolina; Department of Health Behavior, UNC Gillings School of Global Public Health, Chapel Hill, North Carolina; Department of Health Behavior, UNC Gillings School of Global Public Health, Chapel Hill, North Carolina

## Abstract

**Purpose:**

The purpose of this study is to assess the content validity of the Diabetes Distress Scale (DDS) among adults with type 2 diabetes mellitus (T2DM) living in rural Dominican Republic communities.

**Methods:**

Researchers conducted cognitive interviews with 20 adults with T2DM to assess how they answered a Spanish version of the 17-item DDS, a commonly used scale to measure diabetes distress. Interviews were done iteratively to allow for revisions and testing of those revisions with the participants. Analysis involved field notes, text summaries, and cognitive coding.

**Results:**

The sample was 55% women, had a mean age of 55 years, and came from 10 rural communities. The cognitive interviews highlighted needed changes across comprehension, judgment (clarity), recall, response process, and logical/structural issue domains. Participants generally understood the DDS; however, 4 items, the introduction, and response options were revised to improve participant response. The items were revised using wording from the participants themselves. By changing certain terms and splitting a couple of items, these items improved comprehension and judgment. The introduction was simplified from 2 paragraphs to 1 to reduce structural issues (ie, scale’s features), and the response options were reduced from 6 options to 5 options to improve the response process.

**Conclusions:**

Based on iterative study findings, the researchers propose expanding the 17-item DDS to 19 items to improve participant response. Revising the DDS to account for cultural and structural changes will improve clinical and public health understanding of the role of diabetes distress on T2DM management among Dominican adults.

Diabetes distress encompasses a range of feelings, including “frustration, anger, overwhelm, and depressive symptoms as related to the difficulties with self-managing diabetes.”^1^ Diabetes distress has been linked with poor type 2 diabetes-related outcomes for decades.^2^ A recent meta-analysis found that diabetes distress has a negative association with glycemic control, both directly and indirectly, and that 36% of people with type 2 diabetes mellitus (T2DM) report having diabetes distress.^2^

Diabetes distress impacts behaviors associated with T2DM self-management.^3^ A clinical study examining the independent effects of diabetes distress on diabetes-related management behaviors found that greater diabetes distress was associated with lower levels of diet quality, engagement in physical activity, and medication adherence.^4^ Additional studies have found that highly distressed patients were less likely to adhere to medication regimens compared to groups with moderate or no distress.^5,6^ In 2019, the American Diabetes Association and US Preventive Taskforce recommended routine evaluation of diabetes distress, specifically, as part of patient management and called for improved validated diabetes distress screening instruments to help providers address the mental health needs of their patients.^7^

The Diabetes Distress Scale (DDS) was developed to measure distress among individuals with diabetes.^1^ The 17-item scale was originally developed in 4 samples of adults in the United States; 2 in military-affiliated medical centers and 2 in diabetes-specific clinics.^1^ The DDS was then translated to Spanish and validated in several Latin American settings, including Mexico, Central America, Argentina, and Uruguay. A translated version of the DDS to Spanish was found to be a valid and reliable measure to identify distress in adults with T2DM in Mexico.^8^ Evaluation of the translated scale primarily included using content area experts (eg, physicians), rather than cognitive interviewing, to review the translated DDS to assess content validity and assess the scale’s psychometric properties, which performed well.^8^

However, the DDS has not been validated in the Spanish-speaking Caribbean, including the Dominican Republic, where T2DM prevalence among adults is over 10%.^9,10^ Qualitative studies with adults with T2DM in the Dominican Republic have found that diabetes distress is produced by a lack of social support from family and friends,^11^ inability to engage in dietary and physical activity recommendations, limited access to care and medication, experiences with clinical teams, and experiences with different sources of stress.^12^

To obtain valid measurement of the burden of diabetes distress among adults with T2DM in the Dominican Republic, there is a need to understand if and how the Spanish version of the DDS is understood in the Dominican Republic and related settings, such as rural Latin American and Caribbean settings, to identify any necessary revisions. Cognitive interviewing is a research method to qualitatively assess the quantitative scale item’s intent and the respondent’s interpretation.^13^ In addition to assessing scale translations,^14^ cognitive interviewing also provides key insights into how the scale may perform across cultural domains in different global contexts.^15,16^

Specific aims of the study were to examine comprehension of the DDS items in a rural Dominican Republic setting and find ways to revise the scale and improve use of the DDS among adults with T2DM in the Dominican Republic. The purpose of the study was to assess the content validity of a Spanish version of the DDS among adults with T2DM living in rural Dominican Republic communities.

## Methods

### Research Design

Cognitive interviews are a qualitative method used to assess scale and item structure, improve respondent participation, and improve the understanding of the phenomenon being studied.^17^ The authors used one-on-one interviews to understand the phenomenon of interest and how the scale is composed from the perspective of the target population. The 2 major approaches to cognitive interviews are think-aloud and verbal probing methods. Think-aloud is an unstructured qualitative approach, and verbal probing is a semistructured approach that involves asking probing (or follow-up) questions to gain more detailed understanding. Cognitive interviews can inform the qualitative and the related quantitative measurement of a phenomenon, in this case, diabetes distress. To this end, the authors conducted a total of 20 interviews with adults with T2DM using the verbal probing method.^18^ The study protocol was approved by the university institutional review board (No. 19-3029).

### Setting

The authors conducted interviews in person at 1 rural community diabetes clinic from February to March 2020 in the Cibao region of the Dominican Republic. At the time of the study, the clinic served 619 patients across 10 communities. Patients receive free medical consultations, clinical and laboratory exams, medications, and health education on T2DM self-management.

### Sample and Recruitment

Inclusion criteria were being diagnosed with T2DM, being over 18 years of age, enrolled in the diabetes program for at least 1 year, able to speak Spanish, and able to provide informed consent. The authors used purposive sampling to identify eligible participants who could speak about their experiences with diabetes distress. All recruitment occurred in person at the clinic every day for a 3-week period. Because a significant number of patients arrived at the clinic a few hours before the clinic opened, the authors recruited and completed cognitive interviews during these waiting periods to reduce interruptions to clinic flow. All participants were consented verbally.

### DDS

The DDS is a 17-item scale that assesses diabetes distress across 4 domains: regimen, emotional, interpersonal, and health care provider distress.^1^ The DDS focuses on individual-level perceptions of whether or not resources have been available to the respondent over the last month for diabetes self-management. The 17 items of the DDS are scored on a 6-point Likert scale from “not a problem” to a “very serious problem.” The higher the mean item score, the higher the respondent’s levels of diabetes distress. For example, mean scores of 3 or more indicate high distress.

### Cognitive Interviews

Cognitive interviews were done by the first author, who has expertise in conducting qualitative studies with Dominican adults living with T2DM in this setting. This study used a verbal probing method, where the first author read the instructions of the DDS and administered the scale verbally to ensure equity in accessibility (ie, literacy levels).^19^ The first author then read each item 1 at a time, and participants responded to the item using the 6-point Likert scale. Once participants responded to the item, the first author probed to assess comprehension, recall, judgment, response process, and logical issues before proceeding with the next item.^17,19^

**Figure 1.**
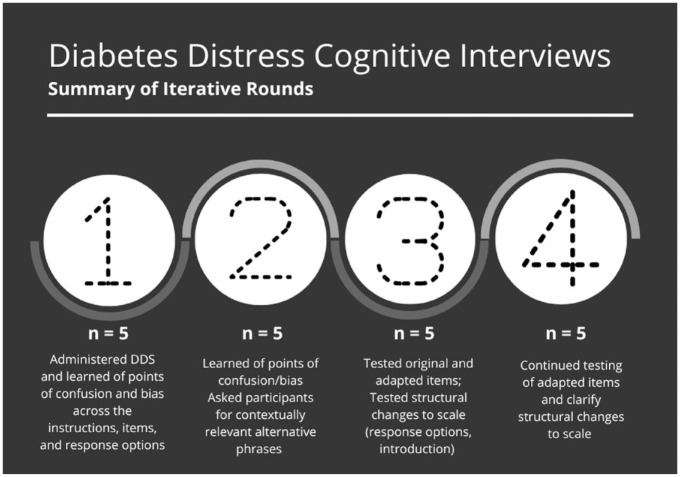
Description of the 4 rounds of cognitive interviews to revise the Diabetes Distress Scale.

The study used a reparative approach to cognitive interviewing. For this, cognitive interviews are done in iterative rounds because the primary purpose is frequently to determine what revisions are necessary for a specific population or setting.^19^ Typically, rounds have no more than 10 participants, and the goal is to assess results, conduct preliminary analyses, make any necessary adjustments to the target items, and continue with additional rounds of recruitment, testing, and revision.^19^ For this study, the first author paused after every 5 interviews to evaluate any needed changes and decide if additional recruitment was needed. The first author conducted 4 rounds of cognitive interviews, interviewing a total of 20 participants (see Figure 1).

**Figure 2.**
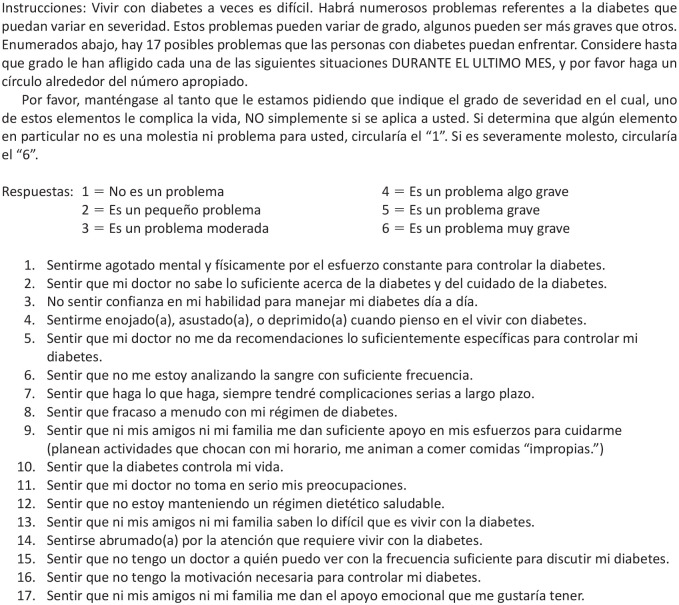
Original Diabetes Distress Scale.

**Figure 3.**
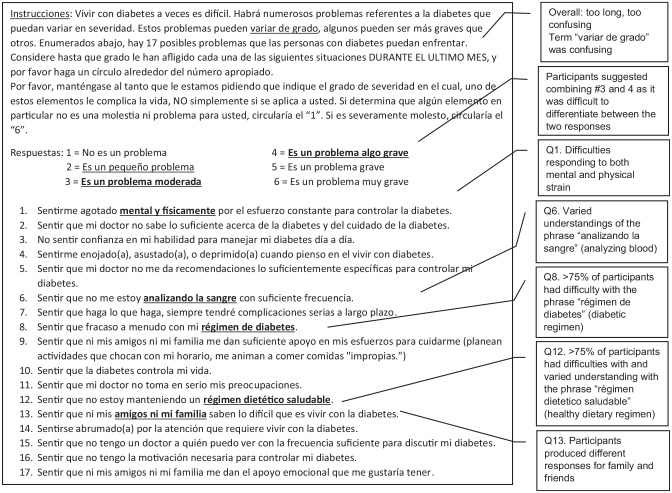
Suggested changes to Diabetes Distress Scale.

**Figure 4.**
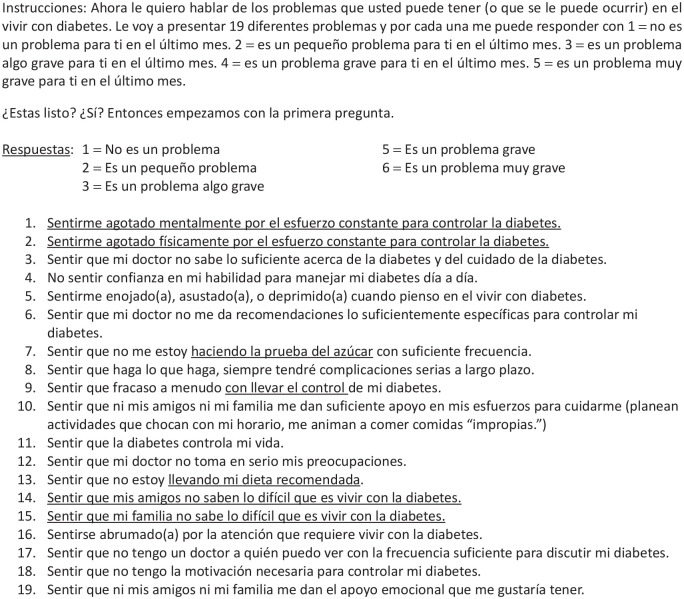
New suggested Diabetes Distress Scale (Dominican Republic).

From the first 5 participants, authors learned of the major points in the scale that were causing confusion and cognitive burden, including the instructions, the response options, and several of the items. For the second set of 5 interviews, adjustments to the instructions made it easier for participants to understand what was asked of them orally. Furthermore, the first author asked participants to provide alternatives to phrases that were causing them confusion to ensure that these items reflected how Dominican adults speak about T2DM self-management. For the following set of 5 interviews, the authors began testing the revised items against the originals to capture perceived ease of response and probe on conceptual understandings of what the items were capturing. In the final round, the first author continued to test the alternatives and followed up on structural changes to the scale, including splitting up items. Using this round-by-round method, the authors were able to make changes in a stepwise fashion and involve participants in sharing their preferences for proposed changes. Changes are presented in Figures 2 to 4.

Interviews lasted 45 to 65 minutes and were audio recorded but not transcribed. Demographic information, specifically age, gender, marital status, and occupation, was collected. All DDS item responses were recorded by hand. The first author took detailed field notes during and after each interview. Audio files (for those who consented to recording; n = 15 of 20), responses, and interview notes were saved physically and electronically according to data safety protocols at the institution.

### Analytic Strategy

Analysis involved field notes, text summaries, and cognitive coding.^19^ The first author completed field notes after each interview, including reactions to the overall cognitive interview, high-level summaries of key themes, and potential areas of coding. As a part of the field notes, the authors developed a matrix recording participants’ ease in responding to each item using a scale of 0 to 2 (0 = very difficult, 2 = not difficult). Second, the authors used a text summary approach to describe dominant themes, conclusions, and problems that were evidenced within the interview notes.^19^ This involved summarizing participants’ responses to each item and any recommendations for item modification. Third, the authors used cognitive coding to code the field notes and text summary across participants according to Tourangeau’s 4-stage model:^17^ (1) comprehension, or the difficulty in understanding the question or remembering it as participants answer, difficulties understanding particular words or concepts, or difficulties due to variation in understanding; (2) recall, or the retrieval of relevant information from memory to respond to the question; (3) judgment or bias, which focuses on the clarity of questions that allow for ease of response, accurately, thoughtfully; and with little fatigue; and (4) response process, or construction of the response options that allows the participant to respond. The authors also coded for logical issues related to the structure of items and the entire scale. The first and senior authors discussed recommended modifications and codes throughout the analysis process. The authors constructed a comprehensive summary across all interviews to identify similarities, determine differences, and finalize suggested changes to phrasing to work best in this setting as well as considerations for similar settings.

## Results

### Participant Characteristics

The authors interviewed 20 participants, all of whom were confirmed to be living with T2DM for at least 1 year. Eleven participants self-identified as women, and 9 participants identified as men. Mean age was 55 years old (range = 31-71). The majority (n = 16) were married or in a union, and the remaining 4 participants were either single (never married) or widowed. Levels of education included primary education (n = 10), secondary education (n = 8), and university education (n = 2). The participants represented 10 rural communities and a range of employment from entrepreneurs, small business owners, school staff, health care staff, technicians, and individuals who worked in their homes. In the following, the results of cognitive interviews across 5 major domains—comprehension, judgment, recall, response process, and logical/structural issues—are presented.

### Comprehension

Comprehension is one of the major areas evaluated when conducting cognitive interviews. Overall, most participants were able to provide their own definitions of concepts and phrases within each item that paralleled what the item was meant to measure. However, a few items were difficult for participants to respond to due to comprehension issues. When asked about “*analizando la sangre*” (analyzing blood [glucose]), 9 out of 20 participants had difficulty responding. When asked how they interpreted the phrase, responses varied; some participants understood that it referred to clinical blood work, but others thought it referred to medications. Several participants could not say what they understood from the phrase at all and, thus, could not respond to the item. When asking participants how they would explain the process of blood glucose monitoring, participants all mentioned preferring an alternative phrase, “*hacienda la prueba del azúcar*” (doing sugar tests), over the original because it was clearer and easier to respond to.

Another item that most participants (16/20) had difficulty understanding was the phrase “*régimen de diabetes*” (diabetes regimen). Participants had a range of interpretations of this phrase, including having T2DM as a moral failure, T2DM as incurable, elevated blood glucose levels, receiving a T2DM diagnosis, and potentially dying from T2DM-related complications. The primary issue was the word *régimen* (regimen). When asked about alternatives, participants brought up phrases like “*control diabetico*” (diabetic control) and “*llevar mi regla*” (follow my rules/recommendations), which get at the same concept but are contextually specific to how diabetes management is spoken about and taught. Ultimately, the preferred alternative and the proposed change to this item is “*llevar el control*” (to keep in control).

The final item that many participants (9/20) did not comprehend was the phrase “*régimen dietetico saludable*” (health dietary regimen). In addition to the issues related to the word “regimen” mentioned previously, *dietetico* (dietary) was difficult to understand. Of those who did understand, they explained it as being aware of what you eat, having an acceptable diet, and having control over one’s diet. But others mentioned that *régimen* meant to correct or that *dietetico* meant to not feel well. Participants suggested an alternative, “*llevando mi dieta recommendada*” (following my recommended diet).

Other items had comprehension-specific concerns; however, they were minor, as was the case with Item 14, where only 3 participants had some difficulty with the word *abrumado*, or overwhelmed. Although the word did slow down these participants’ ability to respond, they were ultimately able to respond to what was asked; thus, the authors left the item as is. In addition, there were points in the instructions that were difficult to comprehend. Discussion of the changes to the instructions are found in the following sections.

### Judgment

Judgment relates to the clarity of items to facilitate the participant’s ability to respond. Items containing 2 components caused major participant response issues. An example of this was Item 1, “*sentirme agotado mental y fisicamente por el esfurezo constante para controlar la diabetes*” (feeling that diabetes is taking up too much of my mental and physical energy every day). Participants understood the item; however, probing revealed that they were often either responding to physical or mental exhaustion or giving distinct severity values for only 1 component. When the authors tested them as separated items, participants gave different response options for each one. Relatedly, one component might be more of a problem than the other; yet when asked together, these numbers were not the average of both physical and mental exhaustion. Given the discrepancy in responses, the authors decided to split the item to make it clearer and easier for participant responses.

Another set of items that had similar issues were items that asked about friends and family (Items 9, 13, 17). For most of these items, emphasizing that the authors were asking about friends and family, not friends or family, was sufficient. All but 1 participant could respond to the original item and respond to the separated items in equivalent ways. The item on emotional support from friends and family followed a similar pattern. However, there was an issue with the following item: “*sentir que ni mis amigos ni mi familia saben lo dificil que es vivir con la diabetes*” (feeling that friends or family don’t appreciate how difficult living with diabetes can be). Participants had an easier time answering it when separated because they sometimes had different answers for friends and family (eg, they may feel supported in one social domain in their life and not supported in the other).

Similarly with the mental and physical exhaustion item, participants did not average out their individual responses when answering the original item; instead, they responded about friends or family support. In addition, the authors probed on who their reference point was for responding, and they mentioned people with T2DM versus not. Given the item’s negative framing, they assumed that they were being asked about friends and family *without* T2DM because “of course they would not understand.” Knowing that participants’ reference point was not just friends or family but, rather, friends or family without T2DM is important in interpreting this item either in its original form or in the proposed split form.

### Recall

The recall period for the scale was 1 month. Recall for participants to address each item was acceptable. Because the scale was applied verbally, the authors repeated to recall period several items throughout the interview to ensure participant responses reflected the relevant time period.

### Response Process

The original set of responses range from 1 to 6 (1 = not a problem, 6 = a very serious problem). Six levels were too many options for participants to remember for a verbal application of this scale. Furthermore, participants found it difficult to differentiate between 3 response options: 2 = *es un pequeño problema* (a slight problem), 3 = *es un problem moderada* (a moderate problem), and 4 = *es un problema algo grave* (somewhat serious problem). Participants mentioned combining either options 2 and 3 or options 3 and 4. After testing these options and probing on preferences for these combinations with 15 participants, the majority of participants preferred combining options 3 and 4. They chose this because the terms *moderada* and *algo grave* felt equal to them in terms of the degree of severity for a T2DM-related concern.

### Logical/Structural Issues

One of the main structural issues was the instructions. The instructions for this scale are about 2 paragraphs in length, which may work for applying the scale for individuals to read on their own, but it does not function as well orally. The instructions required editing to simplify the language and reduce the content to approximately 1 paragraph to ensure participants’ attention could be maintained. One example of text that needed to be simplified was “*variar de grado*,” or the degree to which each of the 17 items distressed them. In addition, after reading the original instructions, several participants believed that they were being asked a question about their T2DM status rather than understanding that each item was a scenario about their potential burdens managing T2DM. This confusion caused participants to respond that their T2DM was a problem or provide anecdotes on living with T2DM. What helped reduce confusion was editing the instructions and adding a question that explicitly clarified to the participants if they were ready to start with the scale.

### Diabetes Distress Elicitation

Participants were asked about sources of diabetes distress in the past 30 days, indicating acute issues. However, all participants responded in ways that reflected their general experiences. For example, participants would use words like *siempre* (always) when responding to questions related to social support, experiences with their providers, and self-management. In the example of providers, because the authors interviewed participants who came monthly, every 2 months, or quarterly to the clinic, they may have needed to average out those visits to respond to what they were asked. However, the authors did not make changes to the 1-month time frame of the scale.

Changes to the scale included separating the mental fatigue and physical fatigue item (Item 1) and separating Item 13 on family and friends not understanding the difficulties with living with T2DM. Items 7, 9, and 13 were adjusted to have clearer, more contextually relevant wording. The response options were reduced from 6 to 5 options. Lastly, the introduction was simplified to improve participant comprehension. The final scale is found in Figure 4.

## Discussion

Diabetes distress is an important factor to assess in the engagement of T2DM self-management behaviors. How individuals engage in T2DM self-management depends on their individualized stressors and resources,^5,20^ and the DDS can provide an understanding of factors that may impede self-management. However, diabetes distress is contextually specific; thus, how DDS is employed in public health research and practice needs to consider how individuals and communities comprehend and talk about this construct. Cognitive interviews provided insight into the functionality of the 17-item DDS in the context of the Dominican Republic. With this method, the authors found that there were items causing cognitive burden among participants and that there was a range of conceptualizations of the phrases used in a Spanish-language version of the scale. These findings suggest that using the existing Spanish-language version of the DDS among this population would likely result in significant measurement error and inaccurate conceptualizations of the constructs purportedly being measured by the scale. Overall, the proposed revisions of the DDS will improve future measurement of diabetes distress among Dominican adults and potentially other Spanish-speaking Caribbean populations.

The authors identified 3 major issues with the scale: comprehension, judgment, and response options. Comprehension issues arose when terms and phrases tested and validated in other regions in Latin America did not transfer to the Dominican context and discourse. Other research findings have indicated that within-language adaption is necessary for participant understanding.^21^ Judgment issues arose with the structure of the item. When participants responded to half of what was being asked or had difficulty responding consistently to a complete item, the best method was to split the item to improve clarity. This reflects survey research best practices of avoiding double-barreled questions because respondents may respond to 1 aspect of the question and disregard the other.^22,23^ Response options concerns have broad implications in scale design. Scale developers must be mindful of the number of response options presented and whether participants can meaningfully differentiate between each response option both in written and verbal formats.

Relatedly, the authors applied the DDS verbally rather than in written form. The implications of this mode were the need to simplify the instructions to ensure participant comprehension. It also related to the number of response options. Six response options, of which several were not easily differentiated, created additional cognitive burden; reducing to 5 response options alleviated participant cognitive burden.

This study provided key areas of revision for the DDS. The qualitative testing of the scale has improved participant experiences across 4 major domains of scale development. Future research should apply this scale in a larger sample to conduct quantitative psychometric testing to assess reliability and validity compared to other Spanish-language scales used in different populations. Evaluation and reevaluation of scale validity and reliability improves at least 2 things. First is ensuring that researchers and practitioners are capturing or accurately measuring the construct that they aimed to measure within the population of interest. Second is ensuring researchers and practitioners are not inadvertently collecting systematically biased data that then can be used to make inaccurate conclusions about a community or population.

This study has several strengths. The study sample was balanced by gender and was comprised of a range of ages and education levels. The study sample of 20 participants was recruited over multiple rounds. Using several rounds of testing revisions and receiving real-time feedback allowed for member-checking of proposed changes. The authors also tested this scale for verbal application to improve accessibility for participants with a range of literacy levels. Limitations of the study are that the sample was pulled from the same geographic region and received care at a diabetes-specific center, which may have influenced how participants experienced diabetes distress. Particularly, health care provider distress may be lower for populations already linked to diabetes-specific care. Future cognitive interviewing studies in the Dominican Republic with people with T2DM who are not linked to care or have access to no- or low-cost diabetes care may provide additional perspectives on diabetes distress.

## Conclusions

Cognitive interviews are a method for scale development and revision that also aids in exploring a phenomenon within a specific context. For this study, the authors gained insight into the content validity of the 17-item DDS in the Dominican Republic and learned more about how the construct of distress manifests in this context. Ultimately, the proposed revised scale included changing phrasing to improve ease of understanding and splitting items to ensure that respondents could provide a focused response to what was asked, thus expanding the original scale to 19 items. The revised DDS can support research to effectively measure diabetes distress among adults in the Dominican Republic and similar settings.
